# Cold atmospheric plasma as an effective method to treat diabetic foot ulcers: A randomized clinical trial

**DOI:** 10.1038/s41598-020-67232-x

**Published:** 2020-06-26

**Authors:** Shahriar Mirpour, Sara Fathollah, Parvin Mansouri, Bagher Larijani, Mahmood Ghoranneviss, Mohammadreza Mohajeri Tehrani, Mohammad Reza Amini

**Affiliations:** 1 Department of Applied physics, Eindhoven university of Technology, Eindhoven, The Netherlands; 2 Department of Applied physics, Amirkabir university of Technology, Tehran, Iran; 3 Skin and Stem Cell Research Center, Tehran University of Medical Sciences, Tehran, Iran; 4 Endocrinology and Metabolism Research Center, Endocrinology and Metabolism Clinical Sciences Institute, Tehran University of Medical Sciences, Tehran, Iran; 5 Diabetes Research Center, Endocrinology and Metabolism Clinical Sciences Institute, Tehran University of Medical Sciences, Tehran, Iran

**Keywords:** Diabetes, Plasma physics

## Abstract

Cold atmospheric plasma (CAP) was shown to decrease bacterial load in chronic wounds. It was also presented as a novel approach to healing wounds in both *in vitro* and *in vivo* experiments. We aimed to examine the first randomized clinical trial for the use of CAP in diabetic foot ulcers. Patients (n = 44) were randomly double-blinded, and assigned to receive standard care (SC, n = 22) without or with CAP, to be applied three times a week for three consecutive weeks (SC + CAP, n = 22), using block randomization with mixing block sizes of four. The trial was conducted at the Diabetes Research Center in Tehran, Iran. CAP was generated from ionized helium gas in ambient air, and driven by a high voltage (10 kV) and high frequency (6 kHz) power supply. Primary outcomes were wound size, number of cases reaching wound size of <0.5, and a bacterial load after over three weeks of treatment. CAP treatment effectively reduced the fraction of wound size (p = 0.02). After three weeks, the wounds to reach fraction wound size of ≤0.5 was significantly greater in the SC + CAP group (77.3%) compared to the SC group (36.4%) (p = 0.006). The mean fraction of bacterial load counted in each session ‘after CAP exposure’ was significantly less than ‘before exposure’ measures. CAP can be an efficient method to accelerate wound healing in diabetic foot ulcers, with immediate antiseptic effects that do not seem to last long.

## Introduction

Diabetes mellitus (diabetes) accounts for 3.9% of annual non-communicable disease-related deaths that occurred worldwide and also caused significant morbidity, impairing the patients’ quality-of-life^1^. Diabetic foot syndrome (DFS) is directly associated with diabetes, contributing to significant morbidity, as well as economic and social burden^2^. It is estimated that approximately 25% of diabetic patients will develop DFS in their lives^3^. Some authors reported that patients have a 3 to 11% annual risk of developing lower-extremity ulcers^2^. Despite optimal treatment, diabetic ulcers are refractory to wound healing, with more than 50% recurring in the wounds after three years^4^. Diabetic foot infection can also lead to complications, e.g., delayed healing process, systemic infections, and amputation. In addition, over 15% of patients with DFS experiences a lower limb amputation^5^. The survival rate is found to be significantly lower in patients who require a lower limb amputation. The cost for two years of care with a newly-diagnosed foot ulcer is over $2,700^5^. This has prompted more research and studies to identify viable alternatives or additive treatment options.

Cold atmospheric plasma (CAP) is an innovative approach in wound healing. *In vitro*^6^ and initial clinical studies for chronic wounds in animals^7,8^ and humans^9–12^ have shown that CAP decreases their bacterial load and promotes healing without any significant side effects on normal tissue. Additionally, CAP was found to facilitate the transformation of the chronic wound from becoming a stagnating wound or an acute healing wound through modulation of the inflammation^13^, stimulation of different growth factors (e.g., those related to angiogenesis/neovascularization)^14,15^, and tissue-reactive species interaction (e.g., nitric oxide (NO), hydroxyl (OH), and atomic oxygen (O))^16,17^. Also, it has been shown that helium CAP treatment decreases the pH in the aqueous medium which may lead to wound acidification, and consequently promotes the healing process^17^. Since these biological issues play a pivotal role in the healing of metabolic ulcers, we recently investigated the effectiveness of this technique on diabetic ulcers in rats. Our results indicate that CAP treatment in diabetic animals accelerates wound healing, leading to the formation of an epidermis layer, neovascularization, cell proliferation, while increasing the release of transforming growth factor **(**TGF)-β1 cytokine from cells in the tissue medium^8^. As such, we will extend our previous findings in humans by conducting a clinical trial to investigate the safety, efficacy, and applicability of CAP treatment for diabetic foot ulcers.

## Results

### Patient characteristics

In total, 22 wound cases were treated by CAP and compared with 22 cases in the control group. Table 1 demonstrates patient demographics, wound characteristics, and history of previous antibiotic use. Most patients had diabetes type 2. All ulcers were grade 2 and mostly located in the forefoot. All patients had a recent history of antibiotic use.

**Table 1:** Characteristics of patients in the study.

Characteristics		Control group	Plasma group
Number of wound cases		22	22
Female		11 (50%)	8 (36.3%)
Average age		54.6 ± 8.8 yr	60.2 ± 5.5 yr
Diabetes type 2		22 (100%)	21 (95.4%)
Diabetes duration		15.7 ± 7.3 yr	19.2 ± 9.4 yr
Smoking		3 (13.6%)	3 (13.6%)
Hypertension		9 (41%)	9 (41%)
Dyslipidemia		9 (41%)	7 (31.8%)
History of previous diabetic foot ulcers		10 (45.6%)	9 (41%)
History of Amputation		2 (9%)	1 (4.5%)
Ulcer on left foot		10 (45.6%)	13 (59%)
Wound location	Forefoot	17 (77.3%)	15 (68.3%)
	Midfoot	2 (9.1%)	6 (27.2%)
	Hindfoot	3 (13.6%)	1 (4.5%)
Ulcer grade: 2		22 (100%)	22 (100%)
Wound type	Neuropathy	17 (77.2%)	19 (86.4%)
	Neuroischemic	5 (22.8%)	3 (13.6%)
Retinopathy or Nephropathy		9 (41%)	6 (27.2%)
Diabetic Mellitus drugs	Oral only	5 (22.8%)	6 (27.2%)
	Insulin only	13 (59%)	10 (45.6%)
	Oral + Insulin	4 (18.2%)	6 (27.2%)
Antibiotics		22 (100%)	22 (100%)
ABI		1.1 ± 0.1	1.1 ± 0.1
FBS		141.8 ± 52.2	164.1 ± 49.7
HbA1c		8.4 ± 1.4	8.8 ± 2

### CAP accelerates wound closure

**Figure 1:**
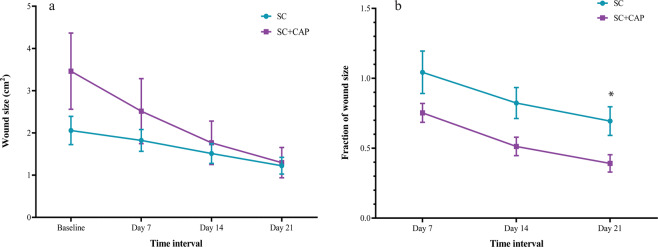
(**a**) wound size in different treatment weeks, (**b**) fraction of wound size in different treatment protocol during treatment time. SC represents standard control group and SC + CAP represents plasma treatment group. *Represents P-value <0.05.

In both SC and SC + CAP groups, the fraction of wound size significantly decreased over time (Table 2, Fig. 1a). Analysis of the data with repeated measure ANOVA showed that while time was significantly effective in decreasing the fraction of the wound size (F (1.2, 51.9) = 30.8, p < 0.0001), there was still a significant difference in the benefit of different treatment protocols (SC = 0.85 ± 0.09 [mean ± SEM] versus SC + CAP = 0.5 ± 0.09; F (1, 42) = 5.4, p = 0.02) (Fig. 1b). In addition, the fraction of wound size was calculated with the log-return to avoid possible statistical error as a function of percentage changes. These results again revealed a significant effect of time (p < 0.0001) and treatment (p = 0.03) in decreasing the fraction of wound size.

**Table 2:** Wound size, Fraction of wound size, and Fraction of bacterial load in different weeks.

	Time			
	*Baseline*	*Day 7*	*Day 14*	*Day 21*
Wound size in cm^2^ (mean ± SEM)				
• SC	2.05 ± 0.33	1.82 ± 0.25	1.51 ± 0.23	1.22 ± 0.19
• SC + CAP	3.46 ± 0.9	2.51 ± 0.77	1.76 ± 0.51	1.29 ± 0.35
Fraction of wound size (mean ± SEM)				
• SC		1.04 ± 0.15	0.82 ± 0.11	**0.69** ± **0.1**
• SC + CAP		0.75 ± 0.06	0.51 ± 0.06	**0.39** ± 0.06*
Fraction of bacterial load (mean ± SEM)				
• SC			10.76 ± 6.15	2.07 ± 0.94
• SC + CAP			2.05 ± 0.91	1.41 ± 0.65

*p < 0.05 compared with SC group.

SEM, standard error of mean; SC, standard care; CAP, cold atmospheric pressure

**Figure 2:**
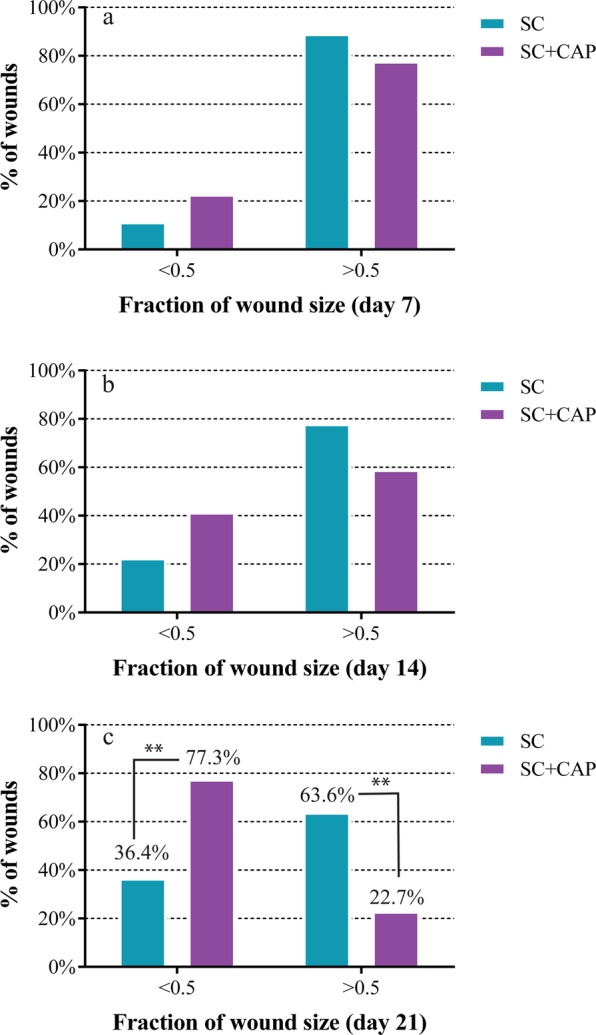
Percentage of wound cases that reached the fraction of wound size of 0.5 after (**a**) day 7, (**b**) day 14, (**c**) day 21. SC represents standard control group and SC + CAP represents plasma treatment group. **Represents P-value < 0.01.

**Figure 3:**
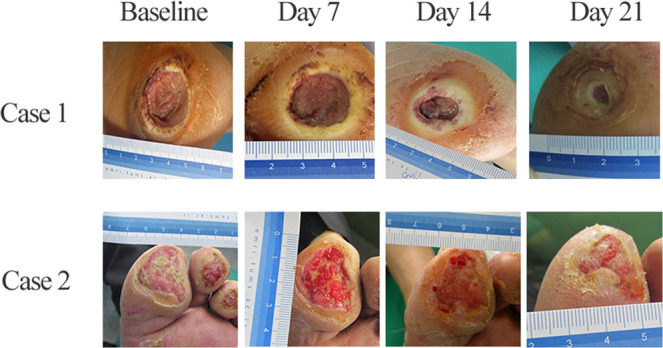
Wound contraction in two plasma treated cases after 3 weeks. Case1: hind foot, case 2: forefoot. SC represents standard control group and SC + CAP represents plasma treatment group.

As an important result of this study, After three weeks, the number of wounds that reached the fraction of wound size at ≤0.5 was significantly greater in the SC + CAP group compared to the SC group (SC = 36.4% versus SC + CAP = 77.3% of total number of cases in each group, chi-square with one degree of freedom = 7.5, p = 0.006). This number was not significantly different between the two groups in the first (p = 0.21) and second (p = 0.19) visits (Fig. 2a–c). In the two treatment groups, the probability of reducing the fraction of wound size ≤0.5 was 5.95 times higher for those with SC + CAP (OR = 0.168; 95% CI = 0.04–0.63). Also, Fig. 3 shows the wound closure process in two cases during three weeks treatment.

### CAP effects on bacterial load

**Figure 4:**
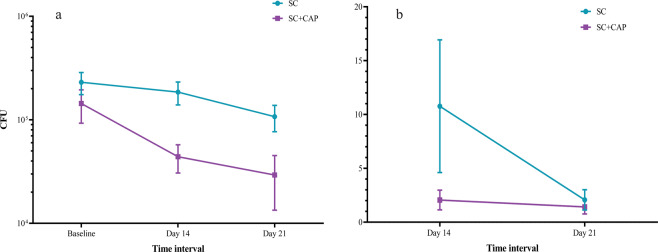
(**a**) CFU reduction in different treatment weeks, (**b**) fraction of CFU in different treatment protocol during treatment time. SC represents standard control group and SC + CAP represents plasma treatment group.

We took bacterial samples before each treatment session. The results of repeated measure ANOVA of log-return showed that time (F (1, 42) = 7.19, p < 0.01) was significant in decreasing the fraction of bacterial load; however, treatment groups did not notably alter the bacterial load (F (1, 42) =1.28, p = 0.26). Moreover, no significant difference was observed in the fraction of bacterial load between study groups at the end of each week (p > 0.05) (Table 2, Fig. 4a,b).

**Figure 5:**
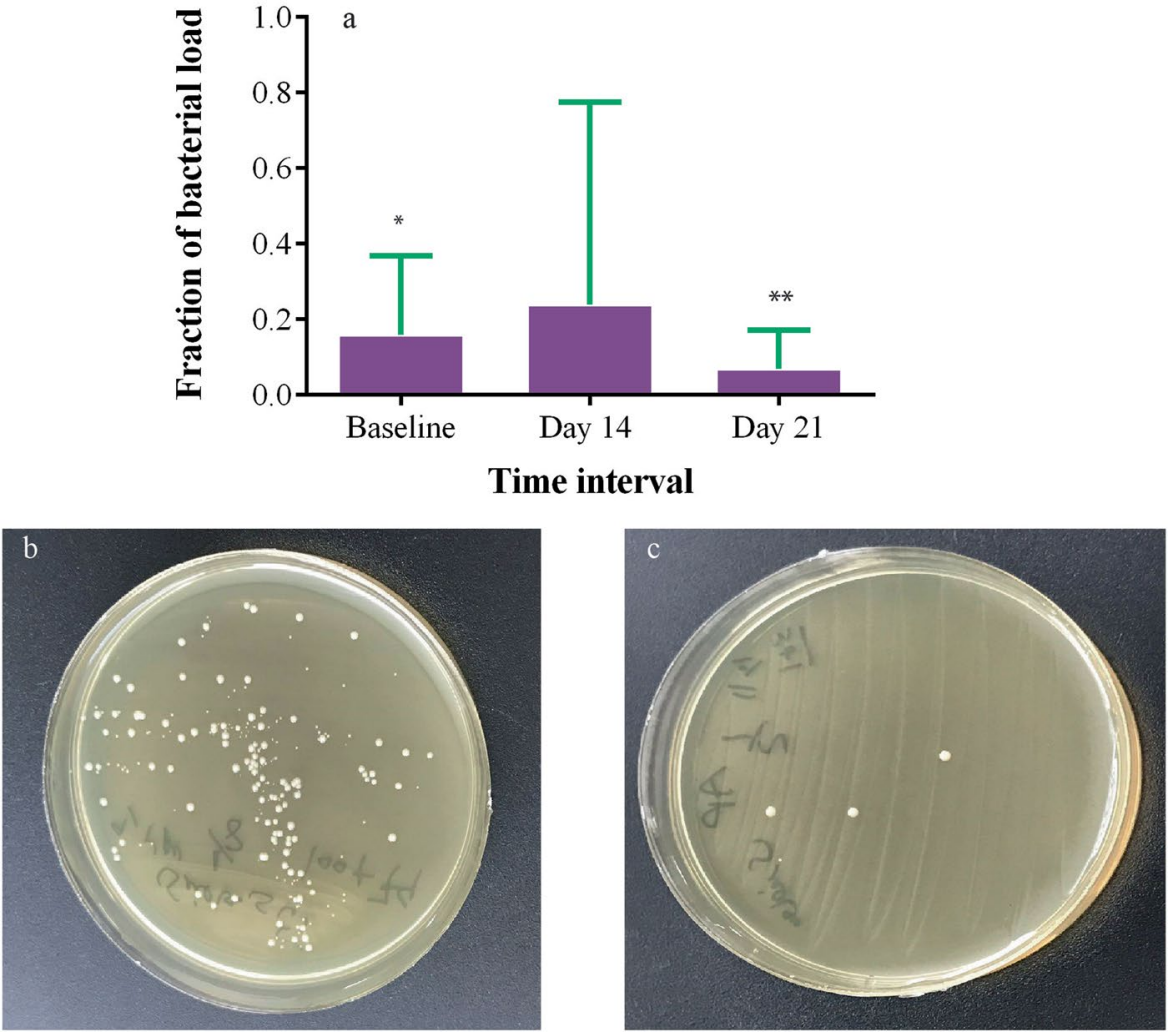
(**a**) Fraction of bacterial load immidiatly before and after plasma treatment at the beginning of each week in the plasma treatment group, Plate dish of cytotoxity test (**b**) before and (**c**) after plasma treatment. *And ** represents P-value < 0.05 and <0.01, respectively.

In order to evaluate the immediate antiseptic efficacy of CAP, the bacterial load was measured before and after each treatment session. CAP treatment led to a significant reduction of the bacterial load at each session in the the entrance and the third week (p < 0.05) (Fig. 5a). Also, Fig. 5b,c show the immediate antibacterial effect of CAP in-vitro before and immediately after 5-minutes CAP treatment. We found no correlation for bacterial load and wound size (week 2: r = −0.1, p = 0.49; week 3: r = 0.06, p = 0.66). Some cases reached the zero value of bacterial load in the first or second week, so were excluded from the statistical analysis the next week.

## Discussion

Our results indicate that three sessions of CAP treatment per week, for three consecutive weeks, accelerate wound healing in diabetic foot ulcers, accompanied by an immediate but brief reduction of bacterial load. To the best of our knowledge, this is one of the few reports that study the effect of CAP in diabetic ulcers. Previous basic and clinical findings also support the effect of CAP in support of wound healing^18^. A starting point for such research is the well-known broad-spectrum bactericidal effect of CAP^18–20^. In this respect, a rich protein medium such as a wound interface is not affecting the efficacy of the direct treatment of CAP^21^. Subsequent basic studies suggest that CAP also directly stimulates the regeneration of damaged tissue on the wound surface^22^. Chronic wounds included diabetic foot ulcers, characterized by wound infections, accumulation of liquid and necrotic tissue, excessive tissue neogenesis, increased release of proteinases and cytokines, and disturbed synchronicity of the different stages of healing^23^. Poor blood supply to the wound bed, as well as neuropathy, exacerbated the healing process in diabetic ulcers^24^. A prolonged inflammatory phase, induced by increased concentration of interleukins and cytokines, delays wound closure^25^. CAP could balance the quality of the inflammatory reaction by decreasing cytokine production and increasing the formation of anti-inflammatory mediators such as IL-10, TGFβ, IL-8^13^. It is assumed that CAP modulates and restores the enzymatically (or non-enzymatically) eliminated nitrogen species (RNS/RONS) or radicals, essential for wound healing^16,26^. CAP also induces cell proliferation and neovascularization^14,15^. However, a selective apoptotic effect of CAP on cytotoxic and T-helper cells, B-lymphocytes, and natural killer (NKT) cells may be involved in regulating the healing process^27^. The CAP in this study can produce a variety of active oxygen and nitrogen products such as O, OH, O_2_^−^, NO, H_2_O_2_. ROS species are beneficial in the eradication of bacterial load. It has been shown that helium CAP can reduce the staphylococcus aureus biofilms up to five orders of magnitude in five minute treatment^28^. We also observed an immediate antiseptic effect after five minute CAP exposure. ROS produced by CAP can induce the intercellular ROS in the bacteria. This accumulation in intracellular ROS can interrupt the cellular function and inactive the bacteria^29^.

Based on these biological premises, clinical trials found the successful treatment of burn wounds, venous ulcers, and chronic ulceration in non-diabetic patients by cold plasma treatment^9–12,30–33^. The assessment of CAP treatment on diabetic wounds was limited mainly to animal studies^8,34^. In our previous paper^8^, we showed that CAP promotes wound healing process in diabetic rat ulcers. It is important to note that there are significant differences between animal models and clinical trials, such as the dependency of treatment efficacy on patient care, different wound shapes or sizes, and different healing mechanisms. Similarly, it was reported that plasma enhances the wound-healing rate in diabetic rodents, presumably due to increased levels of TGF-β1, superoxide dismutase (SOD), glutathione peroxidase (GPx), and catalase (CAT)^8,34^. We show that clinical outcomes in terms of the fraction of wound healing, indicative of the degree of decreased wound size, were statistically lower for the CAP + SC group compared with the SC group. In reviewing previous published studies, accelerating the effect of plasma on wound closure primarily accompanies reduced bacterial load^9–12^. However, in our study, the bacterial wound load was not significantly reduced with CAP three times per week, while the fraction of bacterial load ‘after exposure’ was significantly lower than ‘before exposure,’ with each session indicative of CAP’s short-term antiseptic effect. The high local disinfection quality of plasma treatment immediately after exposure shows that wound bacteria recolonize ten hours after CAP treatment^35^. Patient care was effective in modulating the infection rate of diabetic wounds, which was not addressed here. The frequency and duration of plasma treatments were used more often than the current protocol, according to most previous reports. Isbary *et al*. reported a significant reduction (34%) of bacterial load in 38 chronically infected wounds with a five-minute daily treatment, using the first-generation product of a plasma source, MicroPlaSter α (Hounslow, Middlesex, UK)^19^. They later reported that a two-minute daily treatment with either MicroPlaSter α or the second-generation device, MicroPlaSter β was an effective technique to decrease bacterial load in chronically infected wounds^30^. They found that three to seven minutes of daily plasma treatment for various etiologies over two weeks led to a greater reduction in width and length, compared to controls^12^.

Brehmer *et al*. reported that plasma exposure accelerates wound healing, while it reduces the local bacterial load and pain in patients with chronic leg ulcers, given a treatment protocol of three times per week for eight consecutive weeks^10^. In another study, plasma treatment of pressure ulcers used plasma once a week for eight consecutive weeks, significantly accelerated the reduction rate of wound size, amount of exudate, wound base, and bacterial load. This effect is prominent after one plasma treatment^11^. Antibacterial effects and healing times in other studies can be explained by differences in the time-course of plasma treatment, the plasma operator system, such as the working gas composition, distance or driving power, environmental variables, and biological tissue characteristics^36^. Wound depth, not size^10^, exhibited the major response to CAP treatment, as it is not effective for deep wounds. In light of this, our inclusion criteria were limited to grade 2 ulcers; we also found that wound healing with CAP is independent of the initial wound size.

The current study did have some limitations. The main investigators collecting and analyzing the data were unaware of the assigned treatment, so having the proper placebo for CAP treatment could reduce the possibility of undermining the blinding effect. In our study, patients with diabetic wounds were included, independent of size, infection, or type of colonized bacteria. All aforementioned factors can influence wound healing, although we did not find any significant association between initial wound size and response to CAP treatment.

We treated all wounds with CAP for five minutes per session, irrespective of size: we moved the nozzle in a particular pattern over the wound surface to not leave untreated areas, but it was preferable to adjust the time for wound size to equalize the beam applied to the ulcer’s surface per unit area. This decision was chosen since the wound sizes and shapes were different in each patient and make it challenging to define a protocol for treatment time. It is also notable to state that the treatment time protocol was obtained by the outcome of a limited preliminary study, which we concluded a 5-minute treatment is promising for our research. Safety and risk estimation of plasma treatment have consistently been questioned in medical research. OES shows that most irradiated UV belongs to the 300 to 400 nm, which is not associated with DNA damage^37^. The treatment response and side effects of plasma used for wounds should be assessed for a longer period than three weeks. No specific harm was reported in the study. For a more complete understanding of treatment mechanisms, elaboration of the cellular response after plasma therapy would be desirable.

## Conclusion

The current results show that CAP is an effective method for treating grade 2 diabetic wounds and immediately decreasing the bacterial load. The immediate antiseptic effect associated with the stimulation of cells by CAP may activate the wound healing process. Many questions must be answered: the cell-biology basics of plasma treatment; the appropriate time duration of treatment; and wound characteristics determining the optimal response to plasma therapy. Also, more investigation on the effect of CAP on bacterial response to CAP seems to be needed.

## Material and Method

### Study design

The study was designed as a randomized, parallel, 2-group trial in which eligible patients with diabetic ulcers were randomized 1:1 to receive either standard care of diabetic foot (SC) or SC plus CAP. The study was conducted at the Diabetes Research Center. The study protocol of this trial was registered at IRCT (IRCT20080904001199N2(on 04/07/2018. Ethics committee of the Endocrinology and Metabolism research Centre approved the research protocol and written informed consent was obtained from each patient prior to the study. The study was performed in accordance with the Declaration of Helsinki and the Medical Research Involving Human Subjects Act.

### Patients

**Figure 6:**
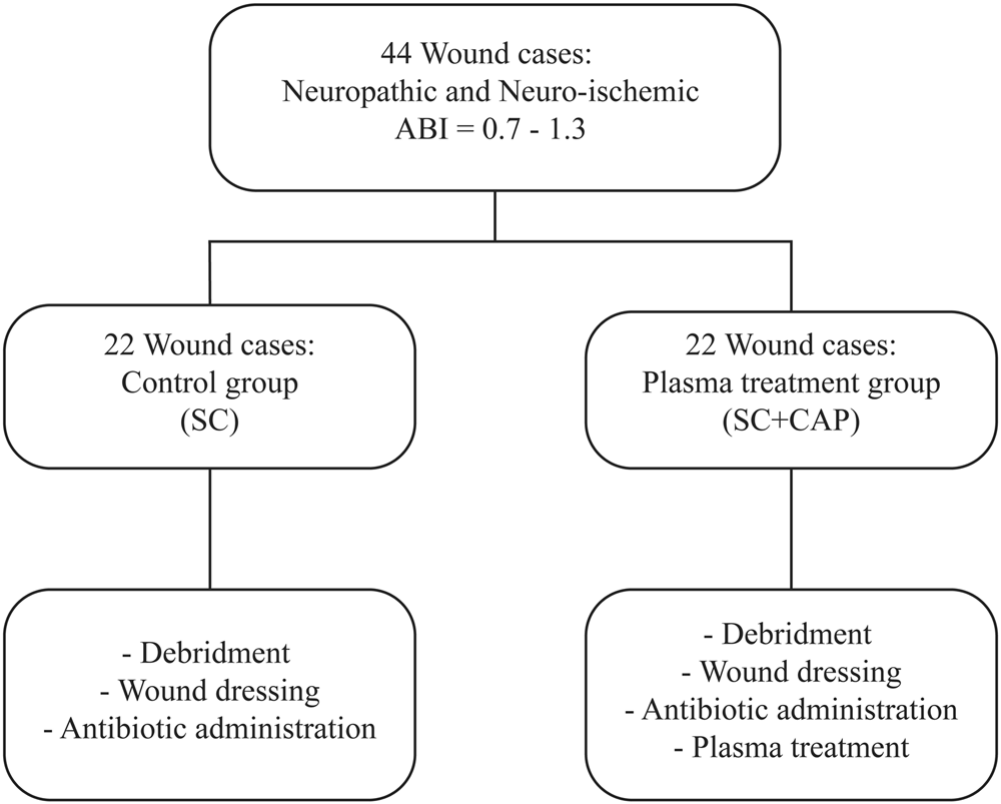
Study groups and treatment descriptions of study protocol.

Of 300 cases who came to the diabetic clinic between September 2016 to September 2017, 44 patients were included in the study (Fig. 6). Participants were eligible for inclusion if they met all of the following criteria: (1) type 1 or 2 diabetes; (2) an ulcer of the lower extremities, graded as Wagner grades 2; and (3) ankle brachial scale (ABI) of 0.7–1.3. Exclusion criteria were (1) age <18 years; (2) history of any cancer; (3) current treatment with chemotherapy or immunosuppressive drugs; (4) pregnancy or breastfeeding; (5) unwillingness of patient. Eligible patients received a full physical examination with documentation of their medical history as well as wound evaluation (number, type, and size). Based on the documentation, we can divide the ulcers into three groups: Neuropathy, Neuroeschemic, and Ischemic. In this study we only investigate Neuropathic and Neuroischemic wounds. Blood was drawn for fasting blood sugar (FBS) and glycosylated hemoglobin (HbA1c) analysis.

### Randomization and blinding

Patients were randomly, double-blind, assigned to an SC group or SC + CAP using block randomization with mixing block sizes of 4. The size of blocks was not disclosed for minimizing the chance of cracking the code. An investigator with no clinical involvement in the trial prepared a computer random number list for block randomization. A trained physician and nurse who were blinded to the randomization method and treatment assignment collected the data. The data also were analyzed by a blinded investigator to the study groups.

### Interventions

All patients enrolled in this trial received standard diabetic foot ulcer care according to the International Working Group on the Diabetic Foot guideline^38^. It included proper antibiotics, glycemic control, and local wound care (intermittent rinsing, dressing, debridement, off-loading, and patient education). Related to the standard care, wounds were cleaned by normal saline. Therefore, all necrotic tissue, foreign debris, bacterial growth, callus wound edge, and wound bed tissue were removed by a surgical blade. Patients in the SC + CAP group treated with CAP moved in a particular pattern over the wound surface in different directions to not leaving untreated wound areas. Each wound was treated for 5 min 3 times a week for three consensus weeks.

**Figure 7:**
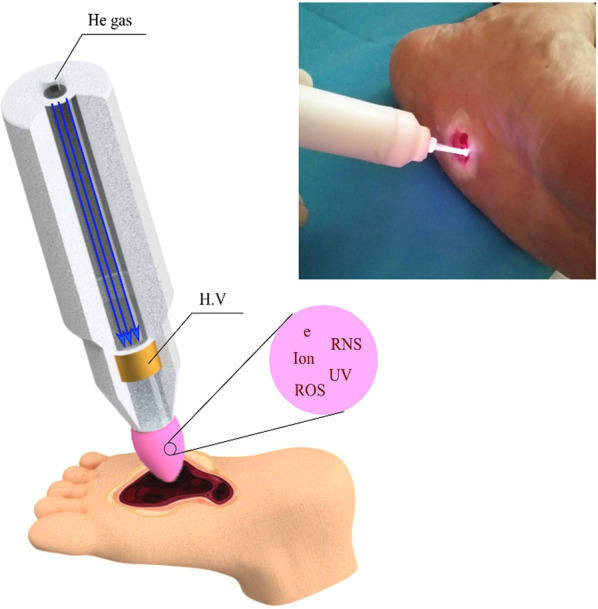
Schematic view and actual helium atmospheric plasma treatment system.

Figure 7 shows the schematic of the plasma setup. The plasma jet consisted of a nozzle made from Pyrex tube (ID: 2 mm and OD: 4 mm). A powered electrode was made from copper with a width of 10 mm and wrapped around the glass tube. Due to the roughness of the tissue surface, we were not able to keep the distance of the nozzle and wound surface constant; however, we tried to keep the distance around 10 mm. The powered electrode was connected to a DC pulsed high voltage (H.V) power supply with a 6 kHz pulse frequency, and 10 kV voltage with a duty cycle of 20%. The input power of the power supply was set to be 30 Watt. The low pulse on-time leads to limit the thermal effect of CAP during the treatment. The feeding gases for this study were 99.99% pure helium (He) with two lit/min gas flow rate. The plasma plum contains reactive oxygen species and reactive nitrogen species, as well as different exited helium states. The optical emission spectroscopy (OES) of the device is given in our previous paper^8^. Since at the time of the study implementation, the device has not received medical approval, all safety regulations were explained to the operators. It can be obtained from OES data that most of the UV radiation bands belong to 300–400 nm, which may have a limited effect on the tissue^37^. The temperature of the CAP plume was measured around 32° ± 2° using rotational temprature measurement of the Nitrogen Second Positive System. Previously in our paper^8^, it was shown that no thermal damage was observed in the histological assays. During the CAP treatment, no thermal damages, and electrical shock were reported.

### Data collection and outcome measures

The wound size and bacterial load were measured and recorded in predefined standard case report forms at the beginning of each week after debridement and before CAP. Primary endpoints were the fraction of wound healing and bacterial load.

The fraction of wound healing was the proportion of wound size at the end of that week to the initial wound size at the entrance week. The 2-D images of wounds were analyzed with ImageJ software to determine the size of the wounds.

Once per week from the second week, two standard bacterial swabs were taken from all participants’ wounds immediately after dressing removal and before any treatment and redressing. Additionally, in SC + CAP group bacterial swabs were taken immediately after the plasma treatment. The end of the sterile swab was rotated into the wound for 5 seconds with low pressure to prevent bleeding. The swab then was placed in a sterile tube containing 1 ml of Trypticase Soy Broth (TSB; SigmaAldrich Co). Afterward, 4 ml of medium poured into 4 sterilized tubes for preparing dilutions of 1/2, 1/4, 1/8, and 1/16. Finally, pathogens were preserved on Tryptic Soy Agar (TSA SigmaAldrich Co) by inoculation loop. The plates were incubated for 24 to 48 hours at 37 °C. The grown colonies were then counted and reported. To calculate colony forming unit (CFU), colonies multiplied in the loop and dilution factor. The fraction of bacterial load expresses the division of calculated CFU at the end of that week divided by calculated CFU at the entrance week.

### Sample size and analysis method

Group sample sizes of 22 in SC group and 22 in SC + CAP group were sufficient to achieve an 80% power to detect a difference of 0.7 between the group proportions. The results were expressed as mean ± standard error of the mean (SEM) of the wound area and bacterial load. Statistical analysis was performed using the student t-test. A P-value < 0.05 is considered significant. When data were not normally distributed, a transformation was performed to obtain normalized data or a non-parametric test was used. To compare the mean of quantitative variables based on qualitative variables through time, paired t-test, and repeated one-way Analysis of Variance (ANOVA) were used. For qualitative variables, the chi-square test was used. To quantify the strength of the association between two events an odd ratio (OR) with the precision of 95% confidence interval (CI) are employed. It should be noted that, P-values < 0.05 (*), P-values <0.01 (**) and P-values < 0.001 (***) were considered as significant
